# Food safety knowledge among 7th‐grade middle school students: A report of a Brazilian municipal school using workshop‐based educational strategies

**DOI:** 10.1002/fsn3.3587

**Published:** 2023-08-06

**Authors:** Maria Aparecida da Ressurreicão Brandão, Maria Elvira do Rego Barros Bello, Manuella Farias de Souza, Maria Rita de Jesus Carvalho, Bianca Mendes Maciel

**Affiliations:** ^1^ Graduate Program in Animal Science State University of Santa Cruz Ilhéus Brazil; ^2^ Department of Exact and Technological Sciences State University of Santa Cruz Ilhéus Brazil; ^3^ Interdisciplinary Degree in Natural Sciences and Technologies Federal University of Southern Bahia Itabuna Brazil; ^4^ Degree Program in Biological Science State University of Santa Cruz Ilhéus Brazil; ^5^ Department of Biological Sciences State University of Santa Cruz Ilhéus Brazil

**Keywords:** foodborne diseases, hygiene education, microbiology, public health, science teaching, scientific literacy

## Abstract

Practical methodologies that include food safety and hygiene education in pedagogical activities are strategies to prevent foodborne diseases (FBDs). Thus, the aim of this study was to investigate the knowledge of 7th‐grade middle school students regarding food microbiology and food safety, and to apply workshop‐based educational strategies that focus on scientific literacy. The students (144) were initially evaluated using a Likert‐scale questionnaire (pre‐intervention, Q0) with ten objective questions on microbiology and food safety. Once the questionnaire was evaluated, interventions were conducted through five science workshops of 50 min, over a period of 5 months. The workshops included educational games, laboratory practices, videos, and lectures that addressed microorganisms that are known to cause the most common FBDs in Brazil. After each workshop, students were asked to express their opinions and understanding of the content through semi‐structured interviews. Six months after the end of the practical interventions, the students completed a second identical Likert‐scale questionnaire (post‐intervention, Q1), and the answers to both questionnaires (Q0 and Q1) were analyzed by calculating the middle rank. The middle rank of Q1 (mean = 0.65 ± 0.13) was 21% greater than the middle rank of Q0 (mean = 0.44 ± 0.16), and statistical significance was observed (*p* = .0135). This demonstrates that new information acquired during the workshops positively influenced learning. We believe that when practical approaches to food safety are included in school education as a priority, the prevalence of FBD will decrease.

## Graphical abstract

This study investigated the knowledge of 7th‐grade middle school students regarding food microbiology and food safety and applied five workshop‐based educational strategies that focused on scientific literacy. The new information acquired during the workshops positively interfered with learning.

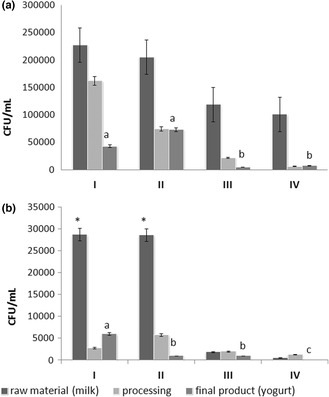

## 1 INTRODUCTION

In Brazil, food and nutrition education is included in the “Law of Food and Nutrition Security No. 11.346” (Brasil, 2006a), which covers policies to sustain life and promote health. Access to safe and nutritious food that is free from physical, chemical, and biological hazards is necessary to maintain these standards (FAO, IFAD, UNICEF, WFP and WHO, 2020). Learning about microorganisms is important to achieve healthy eating practices and disease prevention, and it is not possible to study food safety without an understanding of the microbiology involved. Microorganisms [or pathogens] and foodborne diseases are persistent concerns; therefore, microbiology and food safety should be included from preschool to high school within a food and nutrition education strategy, which would also promote microbiological literacy in the community (Timmis et al., 2019). These subjects involve three basic demands of society: environment, quality of life, and health, all of which are sustainable development goals (United Nations, 2017).

Education is one of the main pillars of controlling foodborne diseases (FBDs). FBDs constitute one of the most prevalent public health problems worldwide, in both developed and developing countries, as it causes high rates of morbidity (around 600 million) and mortality (approximately 420,000) per year, according to the World Health Organization (WHO), and poses a significant impediment to socioeconomic development (WHO, 2015). Of all FBD outbreaks reported in Brazil, 90.5% are of bacterial origin, mainly *Escherichia coli* and *Salmonella* spp. (Brasil, Ministério da Saúde, 2020). Diseases caused by these bacteria occur worldwide and are not restricted to Brazil. According to the WHO, *Salmonella* and *E. coli* rank at the top of the world list of foodborne pathogens (WHO, 2015). In Brazil, along with foodborne bacterial diseases, the protozoa *Toxoplasma gondii*, the etiologic agent of toxoplasmosis, is highly prevalent and causes a threat to public health. In this country, anti‐*T. gondii* antibodies are present in 54%–75% of the population (Souza & Belfort Jr, 2014).

According to data from the Health Surveillance Department (Brasil. Ministério da Saúde, 2020), almost 40% of FBD outbreaks occur when food is handled at home, further reinforcing the urgent need for effective educational strategies. Consequently, given the epidemiological relevance of FBD and the critical need to reduce outbreaks, practical approaches to food safety should be included in schools globally to educate the food handlers of the future (Cunha et al., 2013; Faccio et al., 2013; Haapala & Probart, 2004; Timmis et al., 2019).

The study of microbiology and food safety can also promote scientific literacy, considering the importance of school‐age student education in the construction of scientific knowledge. Savage and Jude (Savage & Jude, 2014) structured a course using microbiological tools to address the question, “How do we reduce the global burden of disease?” This method has proven successful in the advancement of science literacy. Due to the complexity of science fields, practical activities should be intertwined with theoretical teaching to facilitate learning from the early grades and make scientific literacy meaningful for students. This allows them to form elementary meanings regarding the world around them (Lorenzetti & Delizoicov, 2001; Tenreiro‐Vieira & Vieira, 2011). When practical classes have clear objectives, they can expand the abilities of children and adolescents to investigate and construct scientific concepts. In addition, this methodology furthers the teaching of microbiology and food safety in secondary education in a current and practical context, which equips students with scientific methods to solve public health problems.

This study aimed to investigate the knowledge of 7th‐grade middle school students regarding food microbiology and food safety, and to apply educational strategies based on workshops with an emphasis on the most prevalent foodborne pathogens and a focus on scientific literacy.

## 2 MATERIALS AND METHODS

### 2.1 Study area

This study was conducted with 7th‐grade middle school students of the Municipal School of Coaraci, Bahia state, Brazil. According to data collected by the Brazilian Institute of Geography and Statistics (IBGE, 2019), the municipality of Coaraci has a population of approximately 17,500 inhabitants and a human development index (HDI) of 0.613. In 2015, students in the final years of the local education system scored an average of 3.4 on the Brazilian Basic Education Development Index (“IDEB”), placing the city in position 258 of 417 statewide.

### 2.2 Research design

This study was approved by the Research Ethics Committee (CEP/UESC 2568859). Written informed consent was obtained from all participants.

The research participants were 144 students in 7th‐grade middle school aged 12–16 (67 boys and 77 girls), divided into six classes of 20–36 students. This study was conducted between April 2018 and 2019.

First, students were given a questionnaire containing objective questions related to knowledge of food microbiology and food safety, designed using the Likert scale. The Likert scale, or summation scale, refers to a series of statements related to the research object. Students agree or disagree with the statements and report their level of agreement or disagreement (Likert, 1932).

Initially, the students were evaluated using a pre‐intervention Likert‐scale questionnaire, Q0 (Table 1), administered in April 2018. The same Likert‐scale questionnaire was applied 1 year after the start of the research (6 months after the end of the practice interventions) in April 2019 (post‐intervention questionnaire, Q1). The Likert‐scale questionnaire contained ten questions with five alternatives, each of which was assigned the following values based on studies by Hora et al. (2010): A—Totally disagree (0), B—Disagree (0.25), C—Do not agree or disagree (0.50), D—Agree (0.75), and E—Totally agree (1.0). For questions that used inverted items, the values were also inverted.

**TABLE 1:** Questions applied to 7th‐year middle school students to investigate knowledge of microbiology and food safety.

Questions				
Microbiology is the study of viruses, fungi, bacteria, and protozoa. Microorganisms are everywhere. All microorganisms cause diseases. Microorganisms are important in food production. Some microorganisms are edible. Some bacteria cause diseases and others do not. The main reason we wash our hands before eating is to clean our hands. The expiration date ensures food is safe to eat. Canned foods are safe to eat even if the can is swollen (puffed). Diseases can be spread through food and water				
Answers				
Totally disagree	Disagree	Do not agree or disagree	Agree	Totally agree
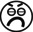	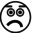	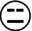	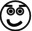	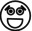

The Cronbach's α coefficient was used to estimate the reliability and consistency of the questionnaire using a scale of 0 to 1. Values ≤0.7 are not considered reliable, while a value of 0.9 indicates that the questionnaire is reliable, thus providing greater robustness to the study (Streiner, 2003).

In the present study, Cronbach's *α* was 0.9, indicating that the questionnaire was reliable for the intended purpose.

Second, a semi‐structured interview with students occurred after the workshops. The interviews were conducted in a manner, whereby the interviewees exposed descriptively what they had learned in the workshops. The answers to the questions in the semi‐structured interviews were recorded, transcribed, and encoded for qualitative analysis. It is important to highlight that students had the complete freedom to answer what they thought was appropriate.

### 2.3 Practical interventions

The practical interventions focused on promoting health and food safety. One month after the application of the pre‐intervention questionnaire, interventions began using five workshops on microbiology and food safety (involving educational games and videos, group sessions, and microbiology experiments) and one lecture. The interventions were conducted in each class involved in the research, during science classes, for 50 min, totaling 5 h in 6 months. After each workshop, students were evaluated using semi‐structured interviews with questions related to each topic addressed in the workshop. Table 2 summarizes the approach, methodology, and form of evaluation of the practical interventions performed during the entire study period. The first and second workshops were conducted sequentially, totaling 1 h and 40 min (50 min for each workshop).

**TABLE 2:** Types of approach, methodology, and form of evaluation of practical interventions carried out in this study.

Period	Apr (2018)	Mai	Jun	Jul	Aug	Sep	Oct	Nov–Mar	Apr (2019)
Approach	*Q0*: Pre‐intervention questionnaire	*Workshop #1*: “The importance of washing our hands” *Workshop #2*: “Microorganisms are everywhere”	School recess	*Workshop #3*: “Storing food in the refrigerator”	*Workshop #4*: “Making yogurt and isolating microorganisms from food handled by students”	*Workshop #5*: “Ball target shooting in food safety and microbiology”	*Lecture*: Toxoplasmosis	Interval	*Q1*: Post‐intervention questionnaire
Methodology	Likert‐scale questionnaire	Educational video and microbiology experiments	School recess	Group dynamic	Microbiology experiments	Educational game	Lecture	Interval	Likert‐scale questionnaire
Form of evaluation	Middle‐rank calculation	Semi‐structured interviews	School recess	Semi‐structured interviews	Semi‐structured interviews	Game score	Observational assessment regarding the participation of students	Interval	Middle‐rank calculation and comparison with the results obtained in Q0

#### 2.3.1 Workshop #1: “The importance of washing our hands.”

Goal: To raise student awareness of the importance of routine handwashing to reduce microorganisms and diseases.

At the start of the workshop, the students learned about the importance of hand washing and the correct way to wash their hands, which was followed by an educational video on the subject. The students were then divided into four groups. The two groups performed hand asepsis by washing with soap and antiseptics with 70% alcohol and imprinting their fingers on nutrient agar plates. The other two groups imprinted their fingers on plates without hand asepsis. The plates were incubated at 37°C for 24 h to allow microorganisms to grow. After the incubation period, the students observed the microbial colonies both macroscopically and under an optical microscope.

#### 2.3.2 Workshop #2: “Microorganisms are everywhere.”

Goal: To highlight the diversity and ubiquity of microorganisms.

Students collected samples from different surfaces (windows, doors, backpacks, notebooks, books, cups, mobile phones, glasses, and parts of their bodies, such as hair, skin, nails, feet, and underarms) using sterile swabs. The samples were cultivated on nutrient agar and incubated at 37°C for 24 h for microbial growth.

After the incubation period, microorganisms were observed under an optical microscope to determine their shape and arrangement. All students used personal protective equipment (cap, gloves, mask, and coat) to prevent contamination by microorganisms.

The first and second workshops were conducted consecutively, over a period of 1 h and 40 min (50 min for each workshop). At the end of these workshops, the students were asked the following questions in the semi‐structured interviews:

- What forms of bacteria did you see under the microscope?
- Why should we wash our hands?

#### 2.3.3 Workshop #3: “Storing food in the refrigerator.”

Goal: To understand the importance of food labels and the position and storage conditions of food in the refrigerator.

At the start of the workshop, the students discussed the following: What is safe food? What is the purpose of food labels?

After a group discussion, the students were shown how to arrange the food in the refrigerator. For this demonstration, three drawings on brown paper of a refrigerator (±1.5 m high) were fixed on a board. The students were divided into three groups and given figures of different types of food (vegetables, fruits, eggs, boxed juice, dairy products, water, and meat products) that they should correctly place in the fictitious refrigerator.

At the end of the session, the correct location for each food item was clarified with the students, and two educational videos of 10 min each, on the subject covered in this workshop, were shown. At the end of this workshop, the students were asked the following questions in the semi‐structured interviews:

- What is a safe food for consumption?
- How should we arrange food in the refrigerator?

#### 2.3.4 Workshop #4: “Making yogurt and isolating microorganisms from food handled by students.”

Goal: To demonstrate the function of microorganisms in the food manufacturing process and understand that food products can spread pathogens when they are manufactured without hygiene practices.

In this workshop, yogurt was prepared by taking a slop back of a previous yogurt in four different ways, using raw milk and boiled milk, handled both aseptically and non‐aseptically, to compare bacterial growth before and after processing. Each type of yogurt was prepared by three groups of students. The samples were microbiologically analyzed using rapid ready‐to‐use bacterial identification tests (Compact Dry®) to identify and count bacteria from the family Enterobacteriaceae, particularly coliforms, *Escherichia coli*, and *Salmonella* spp. Aerobic mesophilic heterotrophic microorganisms were also enumerated using plate count agar (PCA) medium. Food samples (10 mL) before, during, and after processing were suspended in 90 mL of peptone water, and a 10‐fold serial dilution was performed (10^−1^ to 10^−5^). Subsequently, 1 mL of each dilution was inoculated into Compact Dry® ETB plates (for enumeration of Enterobacteriaceae) and the PCA culture medium. For *Salmonella* spp. analysis, diluted samples 1:10 (v/v) in peptone water were pre‐incubated at 37°C for18–24 h before being distributed in the Compact Dry® SL plate. All the plates were incubated at 37°C for 18–24 h. After the workshop, students were asked the following questions:

- What bacteria did we find in the milk samples?
- Name some food products that are produced using bacteria.

#### 2.3.5 Workshop #5: “Ball target shooting in food safety and microbiology.”

Goal: To ensure that the content covered in the workshops was assimilated, and to assess learning.

The students were divided into three groups. One student from each group threw a ball at a target. To earn the score that the ball hit on the target, the team had to answer a question correctly. Otherwise, the score is not awarded. Each group of students was asked to answer five discursive and five optional questions verbally. All questions were related to issues addressed in previous workshops. The team with the highest score was the winner.

The last session was a lecture given by a guest researcher on toxoplasmosis to help students learn about the transmission, pathogenesis, and prophylaxis of the disease. The speaker discussed the subject and showed a 10 min educational video on the transmission of the protozoa *Toxoplasma gondii*.

### 2.4 Analysis of questionnaires and semi‐structured interviews

The questionnaires (Q0 and Q1) were analyzed by calculating the middle rank according to Severo and Kasseboehmer (Severo & Kasseboehmer, 2017). The obtained means were compared using Tukey's test for paired data. Statistical significance was set at *p* ≤ .05. The following strategy was used to obtain the middle ranking:

$$\text{Weighted mean}\;\left(\mathrm{WM}\right)=\sum \left(\mathrm{fi}.\mathrm{Vi}\right)$$

$$\text{Middle}\;\mathrm{rank}\;\left(\mathrm{MR}\right)=\mathrm{WM}/\left(\mathrm{NS}\right)$$

here,

*fi* = observed frequency of each answer to each item.

*Vi* = value of each answer.

NS = number of subjects.

Middle‐rank results that were ≥0.6 were considered satisfactory for learning, while results <0.6 were considered unsatisfactory.

After each workshop, the students attended semi‐structured interviews to evaluate their understanding of the content. The results were tabulated according to the percentage of each answer.

## 3 RESULTS AND DISCUSSION

In the present study, the students were evaluated on six different occasions. A pre‐intervention questionnaire (Q0) was issued at the start of the research to evaluate the previous knowledge of the students regarding food microbiology and food safety. After Workshops 1, 2, 3, and 4, semi‐structured interviews were conducted to assess their learning, totaling three interviews (one interview included both Workshops 1 and 2). The students then participated in a question‐and‐answer game in Workshop 5, which was also evaluated. Finally, 6 months after the end of the practical interventions, the students answered a questionnaire identical to the original questionnaire (post‐intervention, Q1), and the answers to both questionnaires (Q0 and Q1) were analyzed using the middle‐rank calculation.

### 3.1 Analysis of questionnaires

The mean of the middle‐rank values of Q0 (mean = 0.44 ± 0.16) and Q1 (mean = 0.65 ± 0.13) revealed a 21% increase in the second middle rank, with statistical significance (*p* = .0135; Table 3). Due to changes between 2018 and 2019, such as student transfers to other schools, 144 students answered the first questionnaire and 98 students answered the second questionnaire.

**TABLE 3:** Frequency, percentage, and middle rank (MR) of the answers of the pre‐intervention questionnaire (Q0, *n* = 144) and the post‐intervention questionnaire (Q1, *n* = 98).

Questions		Totally disagree	Disagree	Do not agree or disagree	Agree	Totally agree	MR
1. Microbiology is the study of viruses, fungi, bacteria, and protozoa	Q0	38 (26.4%)	6 (4.2%)	22 (15.3%)	78 (54.2%)	0 (0%)	0.49
	Q1	0 (0%)	2 (2.0%)	4 (4.1%)	59 (60.2%)	33 (33.7%)	0.81
2. Microorganisms are everywhere	Q0	46 (31.9%)	16 (11.1%)	17 (11.8%)	62 (43.1%)	3 (2.1%)	0.43
	Q1	1 (1.0%)	2 (2.0%)	11 (11.2%)	52 (53.1%)	32 (32.7%)	0.79
3. All microorganisms cause diseases	Q0	14 (9.7%)	32 (22.2%)	36 (25.0%)	34 (23.6%)	28 (19.4%)	0.45
	Q1	14 (14.3%)	13 (13.3%)	18 (18.4%)	47 (48.0%)	6 (6.1%)	0.45
4. Microorganisms are important in food production	Q0	26 (18.1%)	16 (11.1%)	28 (19.4%)	56 (38.9%)	18 (12.5%)	0.54
	Q1	6 (6.1%)	14 (14.3%)	19 (19.4%)	43 (43.9%)	16 (16.3%)	0.63
5. Some microorganisms are edible (are used to prepare some foods)	Q0	30 (20.8%)	42 (29.2%)	31 (21.5%)	28 (19.4%)	13 (9.0%)	0.42
	Q1	6 (6.1%)	16 (16.3%)	29 (29.6%)	41 (41.8%)	6 (6.1%)	0.56
6. Some bacteria cause diseases and others do not	Q0	31 (21.5%)	20 (13.9%)	26 (18.1%)	55 (38.2%)	12 (8.3%)	0.49
	Q1	3 (3.1%)	7 (7.1%)	4 (4.1%)	52 (52.0%)	33 (33.7%)	0.77
7. The main reason we wash our hands before eating is to clean our hands	Q0	8 (5.6%)	8 (5.6%)	5 (3.5%)	53 (36.8%)	70 (48.6%)	0.21
	Q1	48 (49.0%)	11 (11.2%)	7 (7.1%)	29 (29.6%)	3 (3.1%)	0.68
8. The expiration date ensures food is safe to eat	Q0	5 (3.5%)	6 (4.2%)	7 (4.9%)	40 (27.8%)	86 (59.7%)	0.16
	Q1	51 (52.0%)	3 (3.1%)	11 (11.2%)	32 (32.7%)	1 (1.0%)	0.68
9. Canned foods are safe to eat even if the can is swollen	Q0	31 (21.5%)	39 (27.1%)	17 (11.8%)	30 (20.8%)	27 (18.8%)	0.53
	Q1	17 (17.3%)	5 (5.1%)	17 (17.3%)	52 (53.1%)	7 (7.1%)	0.43
10. Diseases can be spread through food and water	Q0	11 (7.6%)	10 (6.9%)	24 (16.7%)	59 (41.0%)	40 (27.8%)	0.69
	Q1	6 (6.1%)	7 (7.1%)	15 (15.3%)	50 (51.0)	20 (20.4%)	0.68
				Mean of MR Q0		0.44 (±0.16)	
				Mean of MR Q1		0.65 (±0,13)*	

* Statistical significance (*p* = .0135).

In the middle‐rank comparison of each answer to the questions, seven (1, 2, 4, 5, 6, 7, and 8) of the 10 questions had a higher middle rank in Q1 than in Q0, revealing that the new information acquired during the workshops positively affected learning. Furthermore, these findings demonstrate the importance of practical interventions in the acquisition of new knowledge by valuing and constructing meaningful learning through participation and by opening new possibilities for building knowledge between theory and practice.

When students were asked whether all microorganisms caused diseases (Question 3), their answers showed that learning had not occurred after the intervention or they had not understood the question. According to the middle‐rank results, the answers were unsatisfactory (0.45 in Q0 and Q1). In aspects involving health, students in primary schools had difficulty understanding that most bacteria are beneficial (Ballesteros et al., 2018; Zompero, 2009), as they usually associated bacteria with diseases. Nevertheless, in aspects involving biology itself, this information was well consolidated by students in terms of the essential functions of microorganisms for the maintenance of life on Earth.

The participants were asked whether canned food is safe for consumption, even if the can is swollen (Question 9). The middle‐rank analysis of this question (Q0 = 0.53 and Q1 = 0.43) revealed that the workshops did not help the student understand this concept. It is important to emphasize that the participants of this study are low‐income students, and these canned foods are often cheaper options for consumption. One of the main factors influencing the increase in consumption of ultra‐processed foods is the low family income, because these products are often with a lower price on the market than fresh foods (Pinto & Costa, 2021). Therefore, even when the can is swollen and the appearance of the food does not match the spoiled appearance, this population will not stop consuming it, as it may seem like a waste in needy families. So, this unsatisfactory response may also reflect a difficulty in accepting risks to justify consumption.

### 3.2 Analysis of workshops and semi‐structured interviews

In the present study, experimental workshops were included in science classes to support the learning process of students and improve their scientific literacy. The participants were able to familiarize themselves with experimentation, scientific thinking, and the language of microbiology while acquiring meaningful knowledge regarding food safety.

In the scope of elementary school, according to Lorenzetti and Delizoicov (2001), p .8–9, scientific literacy is understood as

“… the process by which the language of natural sciences acquires meanings and constitutes a means for individuals to expand their universe of knowledge and culture, as citizens inserted in society. Therefore, scientific literacy can be initiated when students start school to ensure their insertion in scientific culture based on an interdisciplinary, contextualized, and critical practice.”

Well‐planned experimental classes with articulated goals allow children and adolescents to develop investigative skills, understand and formulate concepts, and reach aspirations through science (Dillon, 2008). Educators must be committed to correlating class content with student development, thus allowing them to become members of society that can reflect and solve common problems (Savage & Jude, 2014; Timmis et al., 2019).

During the activities, the students showed that they were committed, engaged, motivated, and interested in science, probably because the experimental classes had not previously been included at school. It is noteworthy that even students who had repeated the school year and who were disinterested in most curricular activities showed interest in microbiology after the workshops. The lack of motivation among students and teachers in this class was an initial obstacle to experimental interventions. However, during the workshops, the students participated effectively and seemed highly interested and motivated, as shown in the statements below:

I want to be a scientist!

I want to be a scientist just like you, teacher!

The best class I have ever had!

The workshop was great because learning about microorganisms can help prevent diseases.

The workshop was interesting, a better way of learning.

I learned much more in the practical classes than in the theory classes, because watching is easier.

Several researchers have shown that student attitudes toward practical work are positive. They find most of these activities enjoyable, interesting, and unusual, and the new knowledge is understood (Abrahams & Millar, 2008; Cerini et al., 2003; Sharpe & Abrahams, 2020). Therefore, in the present study, the methodology was effective not only in relaying knowledge but also in reaching unmotivated students.

In Workshop #1, most participants (36.7%) indicated hand washing only as a method of preventing food contamination, and they failed to relate it to the prevention of diseases caused by microorganisms. Only 29.6% of the participants partially answered this question correctly, and 33.7% stated that hand washing eliminated all microorganisms (Table 4). Therefore, the objectives of the workshop were not achieved. This unsatisfactory result was likely due to the first and second workshops occurring sequentially, and much of the information was provided at the same time, which possibly made learning more difficult. Scientific literacy in Brazil is still very unsatisfactory; students are not used to approaches such as these workshops. Therefore, when they are challenged in an activity that involves scientific thinking, the learning process still occurs slowly. In order to be able to comply with the curricular planning and also organize the experimental activities, the teacher must have a good schedule, associating the experimental activities with the other theoretical contents fundamental to the teaching of Science (Gomes & Santos, 2018).

**TABLE 4:** Frequency and percentage of answers to the semi‐structured interviews after the workshops.

Workshops	Questions	Answers^a^	%
1. “The importance of washing our hands”	Why should we wash our hands?	Not to contaminate food	36.7
		To remove all bacteria that are present in the hands	33.7
		To prevent bacteria and disease	29.6
2. “Microorganisms are everywhere”	What forms of bacteria did you see under the microscope?	Cocci, vibrios, bacilii e spirilla	34.7
		“Little worms”, “polka dots”, and “little snakes”	33.7
		I do not know	31.6
3. “Storing food in the refrigerator”	What is a safe food for consumption?	It is the food free of bacteria and fungi that cause diseases	39.8
		It is the food without the expiration date	37.8
		I do not know	22.4
	How should we arrange food in the refrigerator?	Place organized and covered food in containers, and meat in the freezer	56.1
		Vegetables should be separated from other foods	7.1
		I do not know	36.7
4. “Making yogurt and isolating microorganisms from food handled by students”	What bacteria did we find in the milk sample?	*Salmonella*, coliforms and *E. coli*	45.9
		Contains several microorganisms, “mumella”, “muela”, “polyform”, and “salmonetas”	23.5
		I do not know	30.6
	Name some food products that are produced using bacteria	Yogurt and cheese	74.5
		Coriander, cabbage, and lettuce	12.2
		I do not know	13.3

^a^ The sentences were grouped according to the most frequent answers.

In Workshop #2, the students investigated the ubiquity and diversity of microorganisms and were asked about the bacterial forms observed under a microscope after the isolation of microorganisms in different niches. More than 34% answered “cocci,” “bacilli,” “vibrios,” and “spirilla” (Table 4). The frequency of these answers was considered satisfactory because it was the first time the participants had observed microorganisms under an optical microscope, and the first time the nomenclature of bacteria had been presented. In Brazil, this content is usually presented in high school (Souza et al., 2006). In contrast, a similar number of participants (33.67%) stated that they had observed “little worms,” “polka dots,” and “little snakes” (Table 2). These responses are also justified since 7th‐grade students are not familiar with the language of microbiology and have difficulty conceptualizing and explaining the microbiological environment in scientific terms, thereby reverting to the use of common wording.

After Workshop #3, almost 40% of the interviewees answered that safe food for consumption did not cause diseases and that it was free of bacteria and fungi (Table 4). This definition complies with the definition provided by the Codex Alimentarius Commission (Codex Alimentarius Commission, 2003), which states that safe foods “do not cause harm to the consumer when it is prepared and/or eaten according to its intended use.” However, almost 38% of the participants in this workshop associated food safety with the shelf life of the product and responded that safe food did not have an expired due date (Table 4). Another issue addressed in the workshop was the best way to arrange food in a refrigerator. The majority of the respondents (56%) answered this question correctly (Table 4). The success of this workshop may be associated with the way the subject was addressed, that is, using educational videos and a game‐like strategy consistent with that of 7th‐grade middle school students. The incorporation of game‐like strategy in the pedagogical practice develops different abilities that contribute to learning, expanding the network of constructive meanings for both children and young people. This social interaction related to the collectiveness of the game can provide greater motivation and improvement in learning (Alegria et al., 2023).

Students often have difficulty understanding that most microorganisms are useful to other species and that only a small group is pathogenic to humans. This topic has been addressed in primary school science classes in an abstract manner. Thus, Workshop #4 aimed to demonstrate the function of microorganisms in the food manufacturing process (in this case, yogurt) and in the process of contamination by pathogenic bacteria. Yogurt was prepared both aseptically and without the hygiene required for food manipulation so that students could compare the bacterial count results and reflect on the importance of hygiene when handling food. In this workshop, we use both traditional culture methods (to count aerobic mesophilic heterotrophic microorganisms) and the ready‐to‐use Compact Dry method (to identify and count Enterobacteria, *Escherichia coli* and *Salmonella* spp.). In this one, the culture medium consists of chromogenic inhibitory enzymes that promote the identification of microorganisms in specific colors. It has high sensitivity and efficiency when compared to conventional methods (Cesarotti et al., 2007), and it can be used for quality control in different food matrices (Lopes et al., 2018).

**FIGURE 1:**
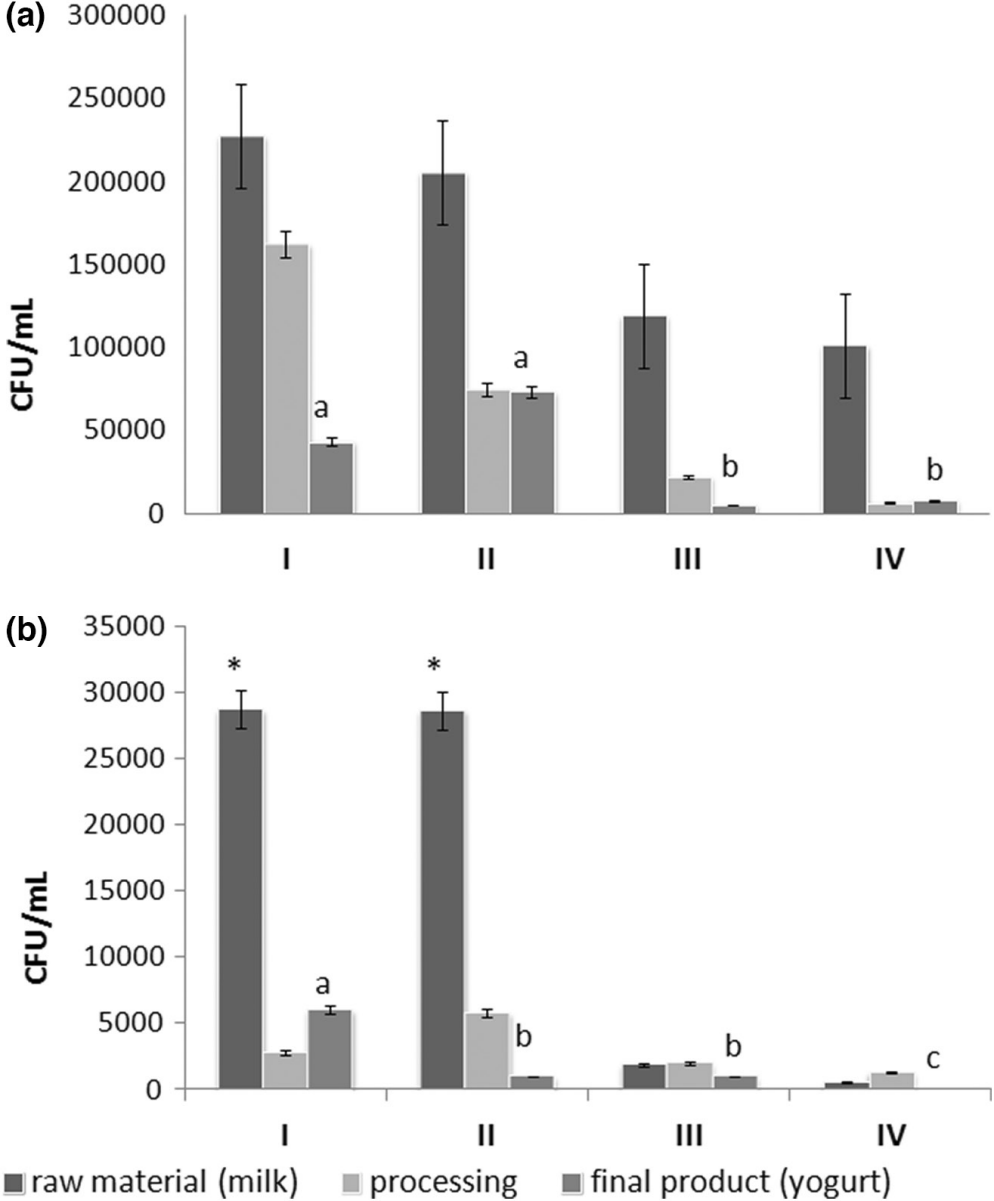
Count of aerobic mesophilic heterotrophic bacteria (a) and Enterobacteriaceae (b) during yogurt processing in workshop #4. Samples (triplicates) were collected in milk (raw material), during processing and in the yogurt (final product). The yogurt was prepared in the following four ways: I—Non‐aseptic production using crude milk; II—Aseptic production using crude milk; III—Non‐aseptic production using previously boiled milk; IV—Aseptic production using previously boiled milk. *Detection of *Salmonella* spp. Different letters mean statistical difference between samples with Tukey test (*p* ≤ .05).

According to the results, the count of aerobic mesophilic heterotrophic bacteria was high in all samples, and during the stages of yogurt processing with and without hygiene it ranged from 5 × 10^3^ to 2.75 × 10^5^ CFU/mL, on average. However, the proportion of this group of microorganisms in the final product (yogurt) was significantly lower (*p* ≤ .05) when prepared with previously boiled milk (Figure 1a). This result enabled an initial discussion with the students about two important points: (i) in milk (and food in general), growth of the broadest spectrum of microorganisms may occur, as in the case of aerobic mesophilic heterotrophic bacteria; and (ii) despite this broad growth, a final product prepared with raw material with lower levels of contamination may have a lower number of microorganisms.

The same result triggered a discussion on the quality of the raw material to emphasize the dangers of consuming unpackaged milk sold in cans on the street. The cans used for selling milk are buckets with lids made of galvanized steel or plastic, with a capacity of 20 L, approximately. The cans are often not properly cleaned, which contributes to the high rate of microorganisms present in the raw material, influencing the quality of the final product. This informal sale of milk in Brazil is prohibited; however, a considerable portion of the municipality's population buys it, as they believe it is a healthier and tastier product, in addition to having a more affordable price. There is no guarantee of the sanitary quality of the product (Trindade et al., 2018).

The milk samples used did not comply with the legal Brazilian standards (Brasil, Mistério da Agricultura, Pecuária e Abastecimento, 2011) of values below 1 × 10^4^ CFU/mL for fresh milk in the standard plate count. Although the consumption of fresh milk can affect consumer health, several factors are used to justify this habit, such as practicality of purchase, lower prices, and the belief that unpackaged milk is healthier than industrialized milk (Bersot et al., 2010; Claeys et al., 2013). In this workshop, students were able to prove, mainly through the Enterobacteriaceae count, that fresh milk could spread foodborne pathogens.

An Enterobacteriaceae count is generally used as a sanitary hygiene indicator to monitor food quality because most of these bacteria inhabit the intestines of people and animals either as members of the normal microbiota or as infectious agents. Therefore, the presence of these microorganisms in the samples analyzed in this study, especially those in raw milk, was important for the discussion on this topic. For the raw material, the average Enterobacteriaceae was 2.86 × 10^4^ CFU/mL, which included the identification of *Salmonella* spp., and 1.20 × 10^3^ CFU/mL in boiled milk. Boiling reduced the Enterobacteriaceae count by an average of 1 log_10_, but without a significant difference, and eliminated *Salmonella* from the sample (Figure 1b). Although the boiling process eliminated *Salmonella*, it was not efficient in eliminating enterobacteria, because when the raw material has a high microbial rate, the processing efficiency for microbiological control may be compromised, especially if it is a homemade process, such as boiling. As it does not go through any quality control, the milk sold informally becomes a public health concern, since it can transmit foodborne diseases, if obtained and handled in inadequate conditions, becoming a potential risk for those who consume it directly, or its derivatives (Trindade et al., 2018). During processing and in the final product (yogurt), a significant decrease (*p* ≤ .05) was observed in the counts of enterobacteria in the samples with previously boiled milk, which highlights the absence of these microorganisms in the yogurt when it was prepared aseptically (Figure 1b). Thus, this practice showed students that microbial contamination of food products can occur at all stages of processing, from the raw material to the final product, thereby stressing the need to use aseptic techniques for handling food. In addition, this workshop introduced the students to practical microbiological analyses conducted in food microbiology laboratories using rapid ready‐to‐use tests to identify and count bacteria.

To assess learning in this workshop, the students were questioned regarding the bacteria detected in milk and yogurt samples. Almost half the students (45.9%) answered correctly and used the nomenclature of microorganisms (“*Salmonella*,” “*coliforms*,” “*E. coli*”). In contrast, approximately one‐quarter of the students (23.5%) answered using other terms (“*mumella*,” “*muela*,” “*polyform*,” and “*salmonetas*”; Table 4) in an attempt to reproduce the microbiological language. In this context, studies conducted by Lorenzetti & Delizoicov (2001) proposed that teaching primary school sciences from the initial grades should encourage students to form and construct elementary meanings of the world around them. It is perceived that the students started to form these meanings during the interventions, and with the encouragement of teachers regarding the development of their investigative curiosity, the students were motivated to build scientific meanings and concepts based on their observations in the workshops, which introduced them to the world of microbiology.

Regarding microorganisms in the food manufacturing process, the vast majority of students (74.5%) were able to understand the importance of bacteria in the production of cheese and yogurt after Workshop #4 (Table 4). A high level of understanding of this topic emphasizes the efficiency of practical approaches. According to Oliveira et al. (2016), only 1.83% of students from the State School of Seridó Paraibano (PB, Brazil) considered the participation of microorganisms in food processing, revealing that this topic is poorly understood by students. Consequently, it is necessary to include food safety education in primary and secondary education (Faccio et al., 2013; Savage & Jude, 2014; Timmis et al., 2019), using simple practical classes and activities.

The educational game “Ball target shooting in food safety and microbiology” was applied in Workshop #5. This game was developed and adapted to assess student knowledge acquired during the workshops. Approximately, 90% of the students answered the questions correctly, demonstrating that they had learned the concepts regarding microbiology and food safety. In recent decades, educational games have been increasingly used as a motivational tool to teach microbiology and food education, thus proposing a stimulating new way for students to learn. Instructional games help students construct knowledge and thoughts and help educators participate in the teaching‐learning process as stimulators and evaluators (Lessa et al., 2020).

A problem that currently prevails in schools is the lack of methodologies for practical application of food and nutrition education and microbiology in pedagogical activities. This is mainly due to the lack of appropriate educational resources, laboratory structures, and time needed by educators to reconcile practice classes, which are work‐intensive, with the theoretical content they must present during the school year. It is important to note that the Ministry of Education and the Ministry of Health, through Interministerial Ordinance No. 1010 (Brasil, 2006b), established that schools, at all levels, are the most appropriate places to apply practical instances of food and nutrition education and microbiology. Learning about microorganisms, their benefits and harms, and how they transmit diseases through food, is critical to promoting food health and safety.

Studies of food safety in primary education have been conducted worldwide. Haapala and Probart (Haapala & Probart, 2004) analyzed 178 high school students in Pennsylvania, USA, and found that 69% of the students interviewed showed an interest in food safety; however, those authors observed a disconnection between knowledge, perception, and behavior related to the subject, and stressed the need for educational practices that motivate food safety in this group. Redmond and Griffith (Redmond & Griffith, 2003) worked with young people in the UK and showed that only 10%–50% of respondents perceived that handling food without proper hygiene leads to a high risk of contamination, and more than 10% of these youths had reported that they had previously experienced an FBD. In another study of 249 children in the 5th year of primary school in Venice, Italy, the authors applied questionnaires and conducted theory and practical interventions on hand hygiene, food handling, and disease transmission and showed that attitudes and knowledge regarding food safety evolved after the interventions (Faccio et al., 2013).

In Brazil, studies like this one involving microbiology and using questionnaires assessments have been carried out with variable results. A study conducted in a public school in the municipality of São Paulo asked 365 participants to complete a questionnaire, after which practical interventions were conducted. The results showed that in the 2 years of research, approximately 35% of the participants evolved in their attitudes toward food safety (Cunha et al., 2013). In another study also conducted in public schools in the municipality of São Paulo with adolescents between 11 and 12 years old, the researchers carried out practical strategies involving microbiology and the students were evaluated through pre‐ and post‐test questionnaires. The results indicated an improvement in knowledge after carrying out the activities and that the microbiology teaching‐learning process is possible even in schools without financial resources to maintain a science laboratory (Cassanti et al., 2008). Bernardi et al. (2019) applied a semi‐structured questionnaire to 87 basic school students from a public school in the state of Rio Grande do Sul, related to bacterial ubiquity. The results were submitted to content analysis, showing that most of students in the early years report microorganisms to dirty places (22.75%), failing to relate them to everyday life, and making microbiology a very abstract topic. Carneiro et al. (2012) applied questionnaires to 317 students of 6th‐ and 7th‐grade classes from three public schools in the city of Aracaju, about the students' perception regarding Microbiology in everyday life. The results showed that although students use basic microbiological knowledge in their daily lives, they were not able to associate science with their daily lives, although they showed that they use this knowledge unconsciously. The authors also demonstrated that the application of alternative Microbiology practices in basic school has promoted gradual changes in students' behavior, directly reflecting on their quality of life, as well as on their better perception of issues in the microbial world.

In this study, we worked to make students aware of the hygiene practices that must be used when handling food, since approximately 40% of outbreaks occur when food is handled at home (Brasil. Ministério da Saúde, 2020). We also alert the risk to public health that unpackaged raw milk sold in cans on the street, can pose. Food sold on the streets, without proper hygiene and inspection practices, can be important sources of pathogenic microorganisms (Khaliq et al., 2021), as well as substances harmful to human health, such as heavy metals (Ali et al., 2022). We believe that prioritizing practical approaches to food safety in primary and secondary schools can be a useful tool to reduce the high prevalence of FBD.

## 4 CONCLUSION

The questionnaires (Q0 and Q1) applied in this research were favorable tools to evaluate student knowledge of food microbiology and food safety before and after interventions. Theory and practical workshops on microbiology and food safety in primary and secondary schools are innovative approaches in Brazil, and it is necessary to include them as teaching methods to support understanding and encourage learning, since most students currently have little knowledge of the microbial world.

## AUTHOR CONTRIBUTIONS

**Maria Aparecida da Ressurreicão Brandão:** Conceptualization (equal); formal analysis (equal); investigation (equal); methodology (equal); writing – original draft (equal). **Maria Elvira do Rego Barros Bello:** Conceptualization (equal); writing – review and editing (equal). **Manuella Farias de Souza:** Methodology (supporting). **Maria Rita de Jesus Carvalho:** Methodology (supporting). **Bianca Mendes Maciel:** Conceptualization (equal); investigation (equal); project administration (lead); supervision (lead); writing – review and editing (lead).

## CONFLICT OF INTEREST STATEMENT

The authors declare no conflict of interest.

## ETHICS STATEMENT

This study was approved by the Research Ethics Committee (CEP/UESC 2568859).

## INFORMED CONSENT

Written informed consent form was obtained from all the participants.

## Data Availability

The data that support the findings of this study are available in the manuscript.
